# GPX3 Overexpression in Cumulus Cells Entails a Poor Prognosis for Uterine Implantation of Morphotype A Embryos

**DOI:** 10.3390/biology11091361

**Published:** 2022-09-16

**Authors:** Ignacio Bejarano, Mónica Dorado-Silva, Helia Sarmiento-Soto, Nuria Álvarez-Sánchez, Patricia Judith Lardone, Juan Miguel Guerrero, Pascual Sánchez-Martín, Antonio Carrillo-Vico

**Affiliations:** 1 Instituto de Biomedicina de Sevilla, IBiS, Universidad de Sevilla, HUVR, Junta de Andalucía, CSIC, 41013 Seville, Spain; 2 Departamento de Bioquímica Médica y Biología Molecular e Inmunología, Facultad de Medicina, Universidad de Sevilla, 41009 Seville, Spain; 3 Ginemed, GeneraLife IVF, 41010 Seville, Spain; 4 Departamento de Bioquímica Clínica, Unidad de Gestión de Laboratorios, Hospital Universitario Virgen del Rocío, 41013 Seville, Spain

**Keywords:** cumulus cells, implantation, GPX3, infertility, transcriptome, oocyte competence

## Abstract

**Simple Summary:**

Despite the fact that human assisted reproduction has undergone extensive development with positive effects on pregnancy, the rates of success are still low. The morphokinetic score of preimplanted embryos is the common routine procedure for ART centres as a reliable predictor of implantation. However, the morphological quality of the embryo is not sufficient to fully ensure implantation success. Therefore, the identification of good predictors of implantation is mandatory to optimise assisted reproduction technology (ART). In this regard, the transcriptome study of the cumulus cells (CCs) is a non-invasive procedure that reflexes the physiological state of oocytes, given the molecular crosstalk between both types of cells. The present study shows a significant down-regulation of the GPX3 gene expression in the CCs isolated from oocytes in pregnant women compared to CCs from non-pregnant women who underwent assisted reproduction. Interestingly, CCs of the highest quality morphotype (A embryos), which achieved implantation success, expressed significantly lower levels of GPX3 expression compared to the embryo morphotype A with implantation failure. Therefore, our observations point to the expression of the GPX3 gene as a potential prognostic marker of bad implantation.

**Abstract:**

Morphological embryo quality is an accurate prognostic tool for the success of assisted reproduction implantation, although complete certainty cannot be guaranteed. The transcriptome of the cumulus cells could be monitored as a faithful reflex of the physiological state of the oocytes, given the molecular crosstalk between both types of cells. Here, we compare the expression of specific genes related to oocyte competence, such as hyaluronic acid synthase 2 (HAS2), cell division control protein 42 (CDC42), connexin 43 (CX43), and glutathione peroxidase 3 (GPX3), in cumulus cells from implanted versus non-implanted embryos in 25 women, using RT-qPCR. After embryo transfer, two cohorts were differentiated: the pregnant group (women with the implantation of 100% of embryos transferred) versus the non-pregnant group (with an absence of embryo implantation), aiming to compare the possible differential expression of the selected genes in the cumulus cells of embryos from each group. HAS2, CDC42 and CX43 did not reveal differential expression between the two cohorts. However, GPX3 showed significantly reduced expression in the cumulus belonging to the pregnant group. Interestingly, even cumulus cells belonging only to morphotype A embryos showed a significantly lower expression of GPX3 in the pregnancy group. GPX3 overexpression in cumulus cells could be a poor prognostic indicator of implantation, discriminating beyond the capacity of the morphokinetic score. Unveiling the cumulus transcriptome could improve successful implantation in assisted reproduction treatments.

## 1. Introduction

In recent decades, people have increasingly demanded assisted reproduction technology (ART). Despite the fact that human assisted reproduction has undergone extensive development with positive effects on pregnancy, the rates of success are still low [1]. Approximately 41% of the embryos transferred from women younger than 35 years achieve successful implantation, while around 33% of the cycles involve delivery [2]. Given the limitation of traditional procedures, the identification of good predictors of implantation is mandatory to optimise ART. In this regard, time-lapse monitoring of preimplanted embryos has allowed us to score and select those with the highest quality, optimising the implantation potential. In fact, embryo scoring has become a common routine procedure for ART centres as a reliable predictor of implantation [3]. Embryo development is scored morphokinetically, taking into account the level of blastomere fragmentation and symmetry, from grade A (the highest implantation prognosis) to grade D (poor implantation prognosis) [4]. Although high-quality embryos show a greater potential for development after vitrification processes [5], embryonic quality is not the only factor that determines a case of infertility, as endometrial receptivity represents a critical factor for embryo implantation [6]. In this way, during assisted reproduction treatments, in which high-quality embryos are transferred, decreased uterine receptivity has been recognised as the basis for numerous cases of infertility [5]. Even with excellent endometrial receptivity, the morphological quality of the embryo is not enough to fully ensure the success of implantation [7]. In this regard, the cumulus cell–oocyte crosstalk is particularly relevant to regulate oocyte maturation [8]. Oocytes strongly depend on cumulus cells (CCs) to perform many metabolic activities, and reciprocally, CCs also need oocyte-secreted factors to avoid differentiation into mural granulosa cells as a default pathway [9]. An increasing number of ions and small molecules have been identified as participants in the bidirectional signalling of cumulus oocytes, such as haemoglobin, long non-coding RNA, cyclic nucleotides, and proteins, through gap junction or exosomal communication [9]. Therefore, the harmonisation of cumulus oocytes is critical for oocytes to achieve developmental competence and become fertilised. An increasing number of studies have shown the potential of CCs transcriptome evaluation to predict embryo competency [10,11] or subsequent aberration [12], receiving special attention beyond the morphological scoring of embryos [13]. Of special interest is the fact that the characterisation of the transcriptomic profile is a non-invasive procedure. In this line, the differential mRNA expressions of several proteins in CCs have been proposed as potential predictors of oocyte competence. In this regard, hyaluronic acid synthase 2 (HAS2) and cell division control protein 42 (CDC42) have been described as good prognostic markers, while connexin 43 (CX43) and glutathione peroxidase 3 (GPX3) were selected among the most remarkable predictors of failure [14,15,16,17]. HAS2 gene expression is promoted by luteinising hormones (LH) in the cumulus–oocyte complex during the ovulation process. It plays an essential role in cumulus enlargement, resulting in proper oocyte maturation [16]. CDC42 is a Rho family GTP-binding protein that plays several cellular functions such as the regulation of apoptosis, transcription activation, cell proliferation and cell polarity. Overexpression of the CDC42 gene in granulosa cells has been associated with competent oocytes that achieved pregnancy outcomes [14]. CX43 plays an essential role in the conformation gap junction, a keystone in CC–oocyte crosstalk, guaranteeing the meiotic arrest and conferring developmental competence [17]. Stressors such as hypoxia act as strong regulators of GPX3 expression, given that GPX3 participates in hypoxia-induced ROS detoxification. GPX3 overexpression has also been associated with ROS production in CCs, oocytes, and follicular fluid [15].

In the present study, our objective was to compare the differential gene expression of HAS2, CDC42, CX43, and GPX3 between CCs belonging to oocytes that produced embryos with a high or low rate of implantation in 25 women.

## 2. Materials and Methods

### 2.1. Subjects

The study was carried out on 25 women undergoing assisted reproduction treatments at the GINEMED Centre for Assisted Human Reproduction (Seville, Spain). To recruit a homogeneous population of patients without severe reproductive problems, the selected participants were healthy women younger than 38 years, undergoing their first or second in vitro fertilisation (IVF) by intracytoplasmic sperm injection (ICSI), or normoresponders with idiopathic subfertility, although patients with a tubal factor or minimal (stage I) to mild (stage II) endometriosis were also admitted. These women also met the inclusion criteria, defined as the number of metaphase II oocytes retrieved greater than three and less than fifteen, representing at least 60% of the total oocytes retrieved. Embryo transfers were performed no later than three months after the date of ovarian puncture, without exceeding the number of three embryos to be transferred, according to the Spanish Law 14/2006 on Reproduction Assisted Techniques. Each subject was determined to be in good health using their medical history and a clinical examination that included routine laboratory tests and screening. Exclusion criteria were patients aged < 18 years, couples with moderate to severe male factor subfertility (according to WHO criteria), polycystic ovarian syndrome (PCOS), poor responders, less than two previous IVF/ICSI cycles, genetic abnormalities, less than four normal fertilised eggs or three transferable embryos, hydrosalpinx, under adjuvant therapies, or unable or unwilling to comply with study procedures. Volunteers were also excluded if they had a record of alcohol abuse in the previous three months or were active smokers. Partial implantation of the embryos transferred per woman was also considered an exclusion criterion, given that it would hamper knowing which embryo failed implantation.

### 2.2. Experimental Design

CCs were divided into 2 groups: the ‘Pregnant group’ were CCs whose 100% transferred embryos were successfully implanted (verified as a positive foetal heartbeat at 9 weeks). In addition, echography discarded monochorionic twinning. The ‘Non-Pregnant group’ were CCs whose embryos transferred were not successfully implanted (0% of transferred embryos were implanted). CCs collected from embryos that produced partial implantation (a lower number of embryos implanted than transferred) were not used in the study to avoid losing track of which of the transferred embryos were successfully implanted. Table 1 shows the baseline value relative to the participants.

Ovarian stimulation was performed following a standardised protocol for all participants. Stimulation was carried out with a dose of 150 IU of recombinant follicle-stimulating hormone (rFSH; GONAL-F^®^, Merck Serono, Frenchs Forest, Australia) and 150 IU of human menopausal gonadotropin (hMG; Menopur^®^; Ferring Pharmaceuticals, Madrid, Spain) on day 3 of the menstrual cycle until the follicles reached a diameter greater than 17 mm. Subsequently, to avoid spontaneous increases, a daily administration of 250 μg of recombinant gonadotropin (0.25 mg of Orgalutran^®^, Organon Ltd., Dublin, Ireland) was administered the day the first follicle reached 14 mm (days 6–7 of the cycle). Stimulation with rFSH and hMG lasted 10 days, and the doses remained unchanged throughout the stimulation protocol. When the oestradiol (E2) was >800 pg/mL and the number of follicles greater than 17 mm was >4, the final maturation of the follicle was induced with a single bolus of 250 μg of human chorionic gonadotropin (hCG) (Ovitrelle^®^, Merk-Serono, Madrid, Spain), 36 h before the follicular puncture. Finally, an average of 10 follicles were recovered by ultrasound-guided transvaginal needle aspiration.

### 2.3. Collection of Cumulus Cells

The denudation of the surrounding CCs was carried out mechanically using two needles and a 1 mL syringe in a fertilisation medium (FM; Sage, Copenhagen, Denmark). Individually, the CCs of each oocyte were then washed in a culture medium (Quinn’s AdvantageTM Medium with HEPES Sage, Copenhagen, Denmark), immediately frozen in liquid nitrogen, and finally stored at −80 °C until analysis.

### 2.4. Sperm Collection and Preparation

The samples were collected by masturbation after 3–4 days of sexual abstinence and then allowed to liquefy for 30 min at 37 °C. Routine seminal parameters were evaluated according to WHO criteria [18]. Briefly, motility was assessed using the computer-assisted semen analysis (CASA) system and verified by an embryologist by a manual method. Sperm concentration and round cell count were determined using the haemocytometer method in two separate preparations of each semen sample, sperm morphology was evaluated by Diff-Quick staining, and sperm vitality was estimated by Eosin-Nigrosin vital staining.

### 2.5. Embryo Classification

Upon ICSI in the egg, fertilised oocytes were cultured and scored on days +2 and +3. The developing embryos were transferred 48 or 72 h after ICSI. The embryos were scored according to the embryo classification criteria proposed by ASEBIR [4] at 16–18 h after insemination (day +1). Such a classification presents different scores to value embryo development according to the morphology. Grade A: equal size blastomeres with less than 10% fragmentation (the best quality embryos and the highest implantation capacity); grade B: equal size blastomeres with a range of 11–25% fragmentation (good quality embryos with high implantation expectancy but not suitable for a single embryo transfer); grade C: different size blastomeres with a fragmentation rate of 26–35% (poor quality embryos with a medium implantation capacity); grade D: includes multinucleated embryos with >35% fragmentation (impaired quality embryos not indicated for transfer).

### 2.6. RNA Isolation, Reverse Transcription, and Real-Time PCR

RNA was extracted from the CCs using TriPure Isolation Reagent (Roche, Mannheim, Germany) according to the manufacturer’s instructions. Single-strand cDNA was synthesised from 1 μg of RNA using the Transcriptor First Strand cDNA Synthesis Kit (Roche, Mannheim, Germany). Real-time PCR was performed on a LightCycler 480 (Roche) using the LightCycler^®^ 480 SYBR Green I Master (Roche, Mannheim, Germany). The primer sequences are detailed in Table 2. All PCR reactions included negative controls in which the template cDNA was omitted. The expression level of each gene was normalised to that of β-actin, and the relative gene expression was calculated using the 2^−ΔΔCt^ method.

### 2.7. Statistical Analysis

All results are expressed as the mean ± standard error of the mean (SEM). Data did not follow a normal distribution, so they were analysed by the non-parametric test one-way analysis of variance followed by Mann–Whitney U tests using SPSS (v25.0, IBM, Armonk, NY, USA). *p* < 0.05 was considered a statistically significant difference.

## 3. Results

No significant differences in age and BMI were observed between pregnant and non-pregnant women (Table 1).

**Figure 1:**
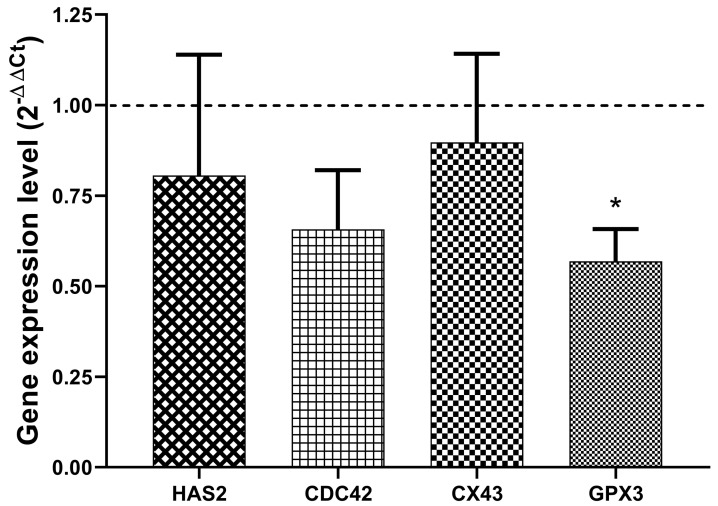
Expression of genes involved in the development of oocytes in cumulus cells. Relative expression of HAS2, CDC42, CX43, and GPX3 in cumulus cells retrieved from follicles of the pregnant group (*n* = 17) compared to the non-pregnant group (*n* = 55; dashed line). Data represent the mean ± SEM. * *p* < 0.001 compared to the non-pregnant group.

The quantification of the relative gene expression of HAS2, CDC42, CX43 and GPX3 in CCs collected from those follicles whose embryos were transferred to women of the pregnant group did show significant downregulation of GPX3 compared to those CCs collected from non-pregnant ones (Figure 1).

**Figure 2:**
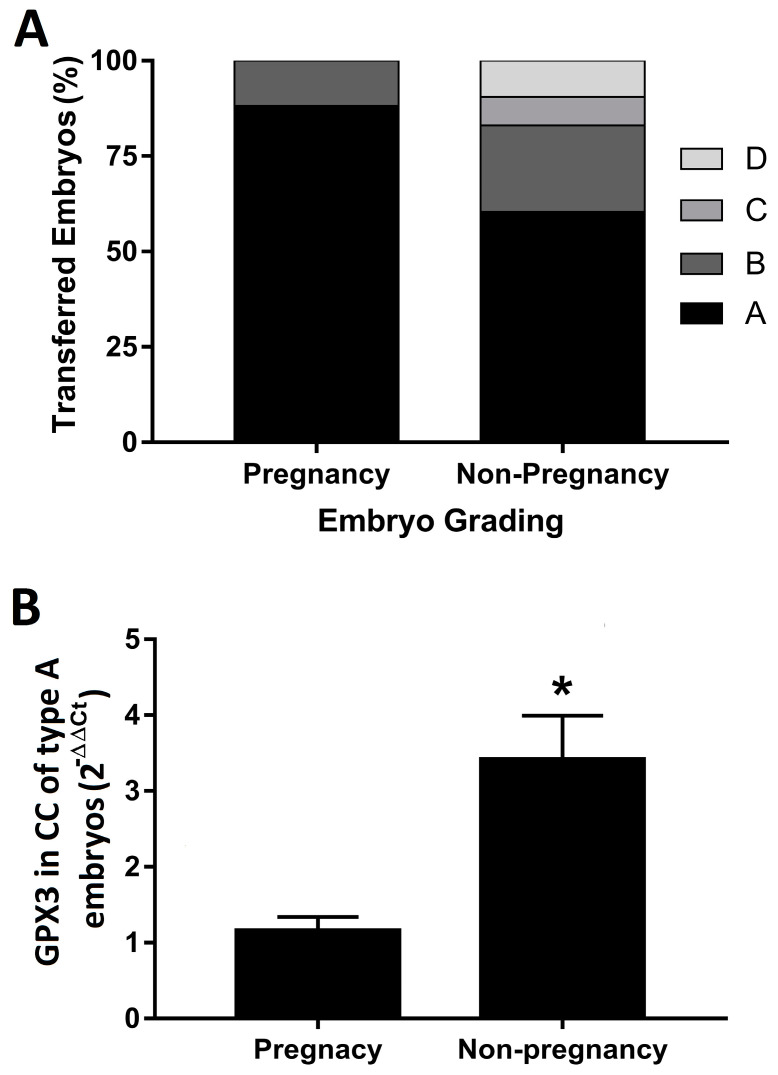
(**A**) Embryo grading. Data represent the % of each grade of embryo quality achieved in both groups for this study. (**B**) Relative expression of the GPX3 mRNA in cumulus cells retrieved from follicles whose embryo achieved grade A quality. Data represent the mean of the calculated values compared to cumulus cells retrieved from follicles of the pregnant group (*n* = 17), with the 2^−ΔΔCt^ method, and the standard error of the mean of each group (n = 55). * *p* = 0.0115 compared to the non-pregnant group.

The grade score of the transferred embryos was of remarkably better quality in the women in whom the embryo implantation was successful (88.2% received an A embryo, whereas 11.8% were transferred with a B embryo). Non-pregnant women received embryos of morphotypes A (60.4%), B (22.6%), C (7.6%) and D (9.4%) (Figure 2A). Interestingly, grade A embryos from non-pregnant women showed a significantly increased expression of GPX3 compared to grade A embryos from the pregnancy group (Figure 2B).

## 4. Discussion

The present study shows a significant down-regulation of the GPX3 gene expression in CCs isolated from oocytes in pregnant women compared to CCs from non-pregnant women who underwent assisted reproduction. Interestingly, CCs of the highest quality morphotype (A embryos), which achieved implantation success, expressed significantly lower levels of GPX3 expression compared to the embryo morphotype A with implantation failure. Therefore, our observations point to the expression of the GPX3 gene as a potential prognostic marker of bad implantation. The expression of the genes studied was independently analysed in other grades of embryo morphotype, such as B (data not shown). However, the limited number of grade B embryos and the lack of morphotypes C and D in the pregnant group (Table 1) made it impossible to study the gene expression pattern in other embryos than those of morphotype A.

A consensus to predict embryo competence for uterine transfer during ICSI-IVF cycles is one of the main challenges in reproduction research. Embryo development is determined by oocyte physiology, which in turn is strongly determined by CC metabolism. CCs are routinely discarded while performing ART; however, they could offer valuable information about oocyte competence through a non-invasive approach, which still aims to be deciphered. The conventional scoring of embryo morphology is the most common non-invasive procedure used as a reliable predictor of implantation [3]. In this framework, understanding the transcriptomic scenarios of CCs and their relationship with the oocyte maturation processes could offer an additional prognostic tool in addition to the morphokinetic analysis in the range of a few (2–3) hours.

CDC42 performs key functions that determine embryo competency [19]. As a factor in oocyte quality, the expression levels of CDC42 in granulosa cells have been positively correlated to pregnancy rates, showing a better prediction of success than follicular fluid antioxidant capacity [14]. It should be noted that, despite the differential expression of CDC42 that can be found between granulosa cells from pregnant and non-pregnant groups, CDC42 transcript levels in granulosa cells do not discriminate significantly between competent (pregnancy) and non-competent oocytes [20]. In this context, no difference was found in CDC42 expression in CCs to discriminate embryos that carried implantation success from failure. Although HAS2 expression in CCs has also been associated with suitable oocyte developmental competence, which met the best morphokinetic characteristics of high-quality embryos for implantation [16], our data do not show significant differences between HAS2 expression levels in the CCs of pregnant and non-pregnant groups. Although several articles have reported a positive relationship between HAS2 expression and fertility [16,21], our data are coherent with previous findings taking pregnancy as the endpoint, in which no association was found between HAS2 levels of CCs and pregnancy [22]. Likewise, CX43 expression correlates with the quantity and quality of the oocyte retrieved, pointing out an improvement in the pregnancy outcome [17]. However, results obtained from human eggs have shown a drop in CX43 levels in CCs once the oocyte reaches the mature stage of metaphase II (MII) [23]. In this regard, the dynamic expression of CX43 has been reported during folliculogenesis, in which CX43 expression levels change during in vitro maturation [24]. Thus, the absence of CX43 expression hampers the progression of folliculogenesis beyond the primary stage. Therefore, the interpretation of CX43 expression as an embryo quality marker implies a particular vision; high levels of CX43 expression in CCs seem a reliable marker related to good expectations of embryo development and reproductive success, while low levels are associated with the arrest of folliculogenesis. However, the dynamic expression of CX43 also presents a range of uncertainty in which these marker levels could change [24]. In the present study, although CX43 levels appeared to show a slight trend to decrease in the non-pregnant group, no significant statistical differences were observed between the groups. In this line, the denudation of follicles performed at different stages of maturation in the present study could represent a confounder and correlate CX43 levels with oocyte competence.

Among the gene markers tested in this study, only GPX3 showed a statistically significant difference in gene expression levels between the pregnant and non-pregnant groups. According to our results, embryo quality development has been negatively related to ROS overproduction, as well as mRNA GPX3 levels [15]. Decreased levels of GPX3 have also been observed in CCs isolated from competent oocytes [25]. It should be noted that our results also show the overexpression of GPX3 mRNA in CCs from the non-pregnancy group compared to the pregnant group, even when only CCs from morphotype A embryos were studied. Hence, the analysis of GPX3 mRNA levels in CCs adds value to the morphological classification of embryos to predict the possibilities of pregnancy success.

In conclusion, the present study shows that CCs of oocytes that achieved pregnancy express low levels of GPX3 mRNA compared to the CCs of oocytes collected from non-pregnant women. Interestingly, CCs that expressed higher levels of GPX3 mRNA, even from embryos with the best morphological classification (morphotype A), were associated with a failure of implantation. Our data point to the gene expression of GPX3 as a possible candidate biomarker of poor embryonic implantation. Therefore, our results suggest a molecular discrimination that might be complementary to embryo morphotyping, leading to a more accurate selection.

## Figures and Tables

**Table 1:** Participants baseline values.

Non-pregnant (0% implantation rate)	**Participants (*n*)**	**Follicles (*n*)**	**Age (Years)**	**BMI (kg/m^2^)**
	18	55	37.4 ± 6.7	26.8 ± 5.3
	**Embryo Transfer Code**	**Transferred Embryos (*n*)**	**Embryo** **Morphotype**	**Implanted** **Embryos (*n*)**
	01	1	D	0
	02	3	A, D, D	0
	03	3	A, A, A	0
	04	2	A, B	0
	05	3	B, B, C	0
	06	2	A, A	0
	07	1	A	0
	08	2	A, B	0
	09	1	B	0
	10	1	A	0
	11	3	A, A, A	0
	12	3	A, A, A	0
	13	2	A, B	0
	14	2	A, A	0
	15	1	A	0
	16	1	A	0
	17	1	C	0
	18	1	D	0
	19	1	B	0
	20	2	A, B	0
	21	2	B, B	0
	22	2	A, A	0
	23	3	A, A, A	0
	24	2	A, B	0
	25	3	A, B, B	0
	26	2	A, A	0
	27	2	D, C	0
	28	2	A, A	0
	29	1	C	0
**Pregnant** **(100% implantation rate)**	**Participants (*n*)**	**Follicles (*n*)**	**Age (years)**	**BMI (kg/m^2^)**
	7	17	37.0 ± 7.2	25.7 ± 2.8
	**Embryo Transfer Code**	**Transferred Embryos (*n*)**	**Embryo** **Morphotype**	**Implanted** **Embryos (*n*)**
	30	2	A, A	2
	31	2	A, B	2
	32	2	A, B	2
	33	3	A, A, A	3
	34	1	A	1
	35	1	A	1
	36	2	A, A	2
	37	2	A, A	2
	38	2	A, A	2

Data on age and body mass index (BMI) are presented as mean ± SD. Embryo morphotype grade A (A); embryo morphotype grade B (B); embryo morphotype grade B (C); embryo morphotype grade D (D).

**Table 2:** Sequences of the primers and real-time PCR conditions used in this study.

Gene		Sequence	ng of cDNA/Well	T° of Annealing
*HAS2*	Forward	5′-ACTTGTGGATGACCTACGAAGCGATTATCACT-3′	120	65 °C
	Reverse	5′-AAACATCTTGGCGGGAAGTAAACTCGAC-3′		
*CDC42*	Forward	5′-GAAAGGCCTAAAGAATGTATTTGACGAAGC-3′	120	58 °C
	Reverse	5′-TGGGCCTTGTCTCACACGAGTGCAT-3′		
*CX43*	Forward	5′-CAGCGACCTTCAAGCAGAGCCAGCAGTCGT-3′	120	65 °C
	Reverse	5′-TGTTGAGTACCACCTCCACCGGATCAAA-3′		
*GPX3*	Forward	5′-TTACACACATGCCTACAGGTATGCGTGATT-3′	120	58 °C
	Reverse	5′-TGGAGAACTGGAGAGAAAGGGTTGTCACT-3′		
*B-ACTIN*	Forward	5′-GGCCGAGGACTTTGATTGCACATTGTT-3′	120	58–65 °C
	Reverse	5′-CCTTAGAGAGAAGTGGGGTGGCTTTTAGGA-3′		

## Data Availability

The data underlying this article will be shared on reasonable request to the corresponding author.
